# Bullet embolism: a rare cause of acute ischaemia

**DOI:** 10.1093/icvts/ivac006

**Published:** 2022-01-28

**Authors:** Nashwan AlAttab, Tariq Wani, Khalid Alomar, Abdullah Alfozan

**Affiliations:** Department of vascular surgery, King Fahad Medical City, Riyadh, Saudi Arabia

**Keywords:** Sharpnel, Embolization, Tamponade

## Abstract

Embolization of a bullet or shrapnel from the heart (left ventricle) to the peripheral arterial circulation is practically unknown. We present a 38-year-old man with no comorbidities who was referred to our centre with a bullet injury to the left side of his chest. The patient complained of mild pain and numbness in his right lower limb. A trauma series was advised. A contrast angiogram of the peripheral lower limbs showed a bullet in the right popliteal artery with no flow in the tibial arteries. A bullet was removed from the distal popliteal artery at its bifurcation with a long thrombus proximal to it. Removal of the foreign body is the widely accepted management, especially when it leads to symptoms like ischaemia or signs of infection, as was the situation in our case.

## INTRODUCTION

Embolization of a bullet or shrapnel from the heart (left ventricle) to the peripheral arterial circulation is practically unknown, although embolization has been reported a few hundred times to occur from the venous circulation to the right ventricle and the pulmonary circulation. This is usually due to an exsanguinating haemorrhage that occurs because of the penetration of a bullet or shrapnel into an artery or heart—resulting in death. However, rarely, because of self-tamponade, the victim may survive and present with either the usual local effects or, rarely, systemic effects like pleural and pericardial effusion, arrhythmias, pulmonary embolism or, as in our case, peripheral ischaemia.

## CASE SUMMARY

**Figure 1:**
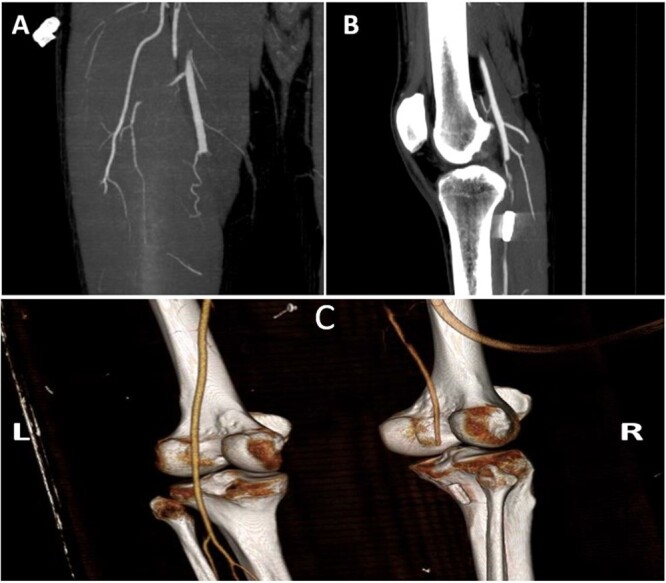
Maximum intensity projection images of computed tomography angiography of the lower limb showing (**A**) total occlusion of the mid and distal segments of the right superficial femoral artery and (**B**) showing occlusion of B2 and B3 segments of the popliteal artery with the bullet distally. (**C**) Volume-rendered computed tomography angiographic images of the lower limbs with a bullet behind the popliteal surface of the right tibia along with occlusion of the popliteal artery proximally.

**Figure 2:**
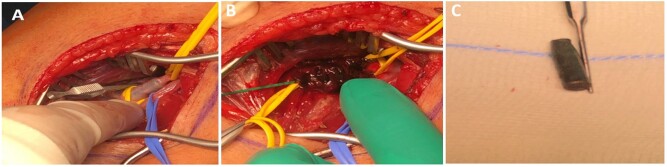
Intraoperative images showing (**A**) exploration of the right popliteal artery followed by (**B**) thrombectomy of the right superficial femoral artery and the popliteal artery and (**C**) the extracted bullet.

A 38-year-old man with no comorbidities was referred to our centre with a bullet injury to the left side of his chest between the 5th and 6th ribs on the left side with a fracture of the 6th rib on the anterolateral aspect. The patient complained of mild pain and numbness in his right lower limb. While the patient was in the primary hospital, he had an echocardiogram that showed pericardial effusion and a computed tomography (CT) angiogram that showed pericardial and pleural effusion. When he arrived at our centre, the patient’s vital signs were stable. His heart rate was 78 beats per minute, his blood pressure was 132/70 mmHg and his temperature was 36.8°C. He showed good saturation on room air (99%). A general physical examination showed an entry wound on the left side of his chest with no exit wound. An examination of his cardiovascular system showed normal heart sounds. His right popliteal pulse and pedal pulses were absent, with no frank signs of ischaemia. A trauma series was advised. X-rays showed a foreign body (bullet-shaped) in the knee area. CT of the chest showed bilateral small pleural effusions with a high density that was suspicious for blood on the left side with atelectasis/consolidation in the lower lobes and lingula. A small fracture with no displacement was noted in the anterior left 6th rib. CT angiogram of the heart, thoracic aorta, abdominal aorta and peripheral lower limbs showed a bullet in the right popliteal artery with no flow in the tibial arteries without evidence of any visceral injury (Fig. 1). The patient was given anticoagulants, and an emergency exploration of the popliteal artery was done. A bullet was removed from the distal popliteal artery at its bifurcation with a long thrombus proximal to it (Fig. 2). The patient was put on an anticoagulatant postoperatively. Follow-up of the patient for the last 9 months showed resolution of his pleural and pericardial effusion and good flow in his right lower limb arteries.

## DISCUSSION

Intravascular embolization of bullets or shrapnel following penetrating vascular trauma is uncommon, with an incidence of 0.3% [1]. It is remarkably uncommon in the upper extremities [2]. Shrapnel fragments have been described to embolize from the peripheral circulation, going to the right ventricle or the pulmonary circulation [3]. Migration down in the venous system under the effect of gravity or to the arterial system through the septal defects can occur [4, 5]. An inconsistent number of entry and exit wounds is indicative of a retained bullet or shrapnel. The possibility of an intravascular location is considered when there is no clinical or radiological evidence of the shrapnel at any place along its course. This situation usually requires a careful examination and multiple investigations. Arterial shrapnel emboli travel distally, becoming lodged at different locations, which leads to end-organ damage or ischaemia.

Removal of the foreign body is the widely accepted management, especially when it leads to symptoms like ischaemia or signs of infection, as was the situation in our case.

## CONCLUSION

Bullet or shrapnel embolization from the heart or great vessels is practically unknown. If it does occur, the best management is the surgical removal of the foreign body with anticoagulation.

## Funding

The study was not funded by any institution or any agency.

**Conflict of interest:** none declared.

## Reviewer information

Interactive CardioVascular and Thoracic Surgery thanks Petre Vlah-Horea Botianu, Kanat Ozisik and the other, anonymous reviewer(s) for their contribution to the peer review process of this article.
